# Antimicrobial resistance in Gram-negative bacteria from retail meat: Prevalence and public health implications

**DOI:** 10.1371/journal.pone.0346434

**Published:** 2026-05-22

**Authors:** Safia Arbab, Hanif Ullah, Weiwei Wang, Anwar Shahzad, Abdullah A. Aseeri, Fuad M. Alzahrani, Khalid J. Alzahrani, Khalaf F. Alsharif, Jiyu Zhang

**Affiliations:** 1 Key Laboratory of Veterinary Pharmaceutical Development, Ministry of Agriculture, Lanzhou, China; 2 Key Laboratory of New Animal Drug Project of Gansu Province, Lanzhou, China; 3 Lanzhou Institute of Husbandry and Pharmaceutical Sciences, Chinese Academy of Agricultural Sciences, Lanzhou, China; 4 Medicine and Engineering Interdisciplinary Research Laboratory of Nursing and Materials/Nursing Key Laboratory of Sichuan Province, West China Hospital, West China School of Nursing, Sichuan University, Chengdu, Sichuan, China; 5 Abbottabad International Medical Institute, Abbottabad, Pakistan; 6 Department of Clinical Laboratory Sciences, College of Applied Medical Sciences, King Khalid University, Abha, Saudi Arabia; 7 Department of Clinical Laboratories Sciences, College of Applied Medical Sciences, Taif University, Taif, Saudi Arabia; Fayetteville State University, UNITED STATES OF AMERICA

## Abstract

Retail meat is an important source of nutrition, but it may also serve as a reservoir of antimicrobial-resistant Gram-negative bacteria, posing a significant public health concern. This study investigated 123 retail meat samples collected from outlets in Sindh, Pakistan, including locally produced raw ground meat, imported raw ground meat, raw beef burgers, frozen chicken portions, and swabs from the outer surfaces of chicken carcasses, to identify Gram-negative bacteria and assess their antimicrobial susceptibility patterns. Gram-negative bacteria were recovered from all samples. Among the isolates, *Escherichia coli* was the most prevalent (37.4%), followed by *Klebsiella* spp. (25.2%), *Salmonella* spp. (17.9%), *Enterobacter* spp. (13.8%), *Pseudomonas* spp. (2.4%), *Citrobacter* spp. (2.4%), and *Aeromonas* spp. (0.8%). Identification was based on culture characteristics, Gram staining, biochemical testing, and API 20E confirmation. Antimicrobial susceptibility testing showed high levels of resistance among the isolates, particularly to ampicillin, amoxicillin-clavulanate, cefotaxime, ceftazidime, and ceftriaxone. *E. coli*, *Klebsiella* spp., *Salmonella* spp., and *Enterobacter* spp. exhibited substantial multidrug resistance, while comparatively lower resistance was observed for cefepime, gentamicin, and piperacillin-tazobactam in some species. Overall, resistance to multiple antimicrobial classes was common, indicating the widespread presence of multidrug-resistant Gram-negative bacteria in retail meat. These findings highlight the potential role of retail meat as a source of antimicrobial-resistant bacteria in the food chain and underscore the need for strengthened surveillance, improved hygiene practices, and antimicrobial stewardship in food animal production. A One Health approach is essential to help limit the spread of antimicrobial resistance across human, animal, and environmental sectors.

## 1. Introduction

Antimicrobial resistance (AMR) is a critical global health concern, directly responsible for an estimated 1.27 million deaths per year and contributing to nearly 5 million deaths worldwide [1–3]. Gram-negative bacteria, such as *Escherichia coli*, *Salmonella* spp., and *Campylobacte*r spp., pose significant challenges due to their ability to acquire and distribute resistance genes via mobile genetic elements, including plasmids and integrons. These bacteria are major contributors to foodborne illnesses, urinary tract infections, and septicemia, and their resistance considerably limits treatment options [4–6].

Retail meat products, including beef, turkey, and chicken, can become contaminated with bacteria during transport, processing, or slaughter. Such contaminated meat serves as a pathway through which humans may be exposed to antimicrobial-resistant bacteria arising from the use of antibiotics in food animals. Consumption of meat carrying resistant bacteria can directly cause foodborne illness or contribute to the spread of resistance by transferring resistance genes to bacteria in the human gut, potentially leading to infections that are more difficult to treat [5,7–9].

Surveillance programs, such as the U.S. National Antimicrobial Resistance Monitoring System (NARMS) and the European Centre for Disease Prevention and Control’s (ECDC) European Antimicrobial Resistance Surveillance Network (EARS-Net), have consistently identified antimicrobial-resistant bacteria in retail meats [10,11]. Notably, high levels of fluoroquinolone resistance have been observed in *Salmonella* and extended-spectrum beta-lactamase (ESBL)-producing *E. coli*, as well as in *Campylobacter* spp. from poultry. Multidrug-resistant strains are frequently most prevalent in poultry, particularly chicken and turkey, although rates vary by region and meat type [12–14]. This paragraph focuses on resistance prevalence, while specific mitigation or control strategies are discussed elsewhere in the manuscript.

Additionally, plasmid-mediated resistance genes, including ESBL/AmpC enzymes and *the mcr gene (associated with colistin resistance), have been identified* in isolates from retail beef in recent studies [15]. The use of third-generation cephalosporins and colistin in human medicine is particularly concerning, as they are considered last-resort antibiotics. Even though the overall prevalence of this resistance remains low in retail meats in the United States and Europe, occasional detections underscore the importance of ongoing surveillance [16,17].

The implications for public health are substantial. Meat containing resistant Gram-negative bacteria can prolong and increase the incidence of food-borne diseases, reduce the availability of treatments, and help AMR spread more widely in communities [18,19]. A One Health approach, less antibiotic use in food animals, better processing cleanliness, increased surveillance, and consumer education regarding safe meat handling and preparation are all necessary to address this issue [20].

Despite global attention to AMR in foodborne pathogens, data from Pakistan remain scarce and fragmented, with most previous studies focusing on clinical isolates or limited sample types. The current study addresses this gap by providing an updated assessment of antimicrobial-resistant Gram-negative bacteria isolated from retail meat across different regions of Pakistan. By examining the phenotypic resistance patterns of Gram-negative bacteria isolated from retail meat, our work offers new insights into the potential role of retail meat as a reservoir and transmission pathway for AMR within the Pakistani context.

## 2. Materials and methods

### 2.1. Ethics statement

This study involved retail meat samples obtained from commercial outlets and did not involve live animals or human participants. Ethical approval was obtained from the Animal Administration and Ethics Committee of the Lanzhou Institute of Husbandry and Pharmaceutical Sciences, Chinese Academy of Agricultural Sciences (Approval No. CARS-37).

### 2.2. Sample collection and study design setting

This study was conducted to investigate antimicrobial resistance patterns in Gram-negative bacteria isolated from retail meat products in Sindh, Pakistan. A total of 123 meat-related samples were collected from retail outlets located in different areas of Sindh province. Retail outlets, including local butcher shops, supermarkets, and meat vendors, were selected based on the availability and accessibility of meat products commonly purchased by consumers. Efforts were made to include outlets from different locations to improve geographic representation and reduce potential sampling bias.

The collected samples included locally produced raw ground meat (n = 31), imported raw ground meat (n = 31), raw beef burgers (n = 31), frozen chicken portions (n = 15), and swabs from the outer surfaces of chicken carcasses (n = 15).

Each sample was aseptically placed into sterile sampling bags (Whirl-Pak®, Nasco, Fort Atkinson), labeled, stored in an insulated icebox, and transported to the laboratory under cold-chain conditions to maintain sample integrity. Sample handling followed established procedures [5]. For microbiological examination, all specimens were inoculated onto MacConkey agar plates (Oxoid, UK) and incubated at 37 °C for 24 hours.

### 2.3. Screening and microbiological analysis

#### 2.3.1. Pre-enrichment and selective culturing.

From each purchased meat sample, 25 g was aseptically weighed and transferred into 225 mL of buffered peptone water (Oxoid, Basingstoke, UK). The samples were homogenized using a sterile stomacher and incubated at 37 °C for 18–24 h for pre-enrichment, following procedures described by the International Organization for Standardization [21]. After enrichment, aliquots were streaked onto selective media: Xylose Lysine Deoxycholate (XLD) agar (Merck, Darmstadt, Germany) for *Salmonella* spp., Cetrimide agar (HiMedia, Mumbai, India) for *Pseudomonas aeruginosa*, and MacConkey agar (Difco, Detroit, MI, USA) for general Gram-negative bacteria enumeration. Additional selective media were applied as required for the isolation of other Gram-negative bacteria. Presumptive colonies were purified by repeated sub-culturing and further characterized using standard biochemical tests and identification protocols [22].

#### 2.3.2. Sub-culturing and identification.

Gram stain and biochemicals, triple Sugar Iron (TSI) oxidase, catalase, citrate, urease, and indole were utilized for the first identification. In accordance with the manufacturer's instructions, species confirmation was carried out using API 20E.

**Gram’s staining:** Gram staining was performed following the standard protocol recommended by the American Society for Microbiology (ASM) [23].

### 2.4. Biochemical tests/IMViC test

#### 2.4.1. Indole test.

Pure bacterial cultures were inoculated into tryptophan broth (HIMEDIA®, Ref: M1339-500G) and incubated at 37 °C for 24 hours. After incubation, 2–3 drops of Kovac’s reagent were added, and the reaction was examined after 15 minutes. A red ring at the surface indicated a positive result, while no color change indicated a negative result [24].

#### 2.4.2. Methyl Red (MR) test.

MR-VP broth (HIMEDIA®, Ref: M070-500G) was inoculated and incubated at 37 °C for 24 hours. After incubation, 2–3 drops of methyl red indicator were added. A stable red color indicated a positive result, showing that the organism performed mixed acid fermentation. A yellow color indicated a negative result, meaning insufficient acid was produced to lower the pH [25].

#### 2.4.3. Voges–Proskauer (VP) test.

Cultures were inoculated into VP broth (HIMEDIA®, Ref: M070F-500G) and incubated at 37 °C for 24 hours. After incubation, 2–3 drops of Barritt’s reagent (α-naphthol and KOH) were added. A pink to red color after 15 minutes indicated a positive result, while no color change indicated a negative result [25].

#### 2.4.4. Citrate utilization test.

Simmons citrate agar slants (HIMEDIA®, Ref: M099-500G) were streaked with bacterial isolates and incubated at 37 °C for 24 hours. Growth with a blue coloration of the slant indicated a positive citrate utilization, while green with no growth indicated a negative result [26].

#### 2.4.5. Triple sugar iron (TSI) test.

Bacterial isolates were inoculated on TSI agar slants (HIMEDIA®, Ref: M021-500G) by streaking the slant and stabbing the butt, followed by incubation at 37 °C for 24 hours. A yellow slant and butt indicated glucose, lactose, and sucrose fermentation; a red slant and yellow butt indicated glucose fermentation only; blackening indicated H₂S production; and cracks or bubbles indicated gas formation [24].

#### 2.4.6. Urease test.

Christensen’s urea agar (HIMEDIA®, Ref: M112-500G) was inoculated with bacterial isolates and incubated at 37 °C for 24 hours. A pink coloration of the medium indicated a positive urease reaction, while no color change indicated a negative result [27].

### 2.5. Antimicrobial susceptibility

Antimicrobial susceptibility testing. Each bacterial isolate was adjusted to a 0.5 McFarland turbidity standard and evenly spread onto Mueller-Hinton agar plates using a sterile swab disc diffusion technique, based on Clinical Laboratory Standards Institute (CLSI) recommendations [28]. Each bacterial isolate was evenly spread on separate nutrient agar plates, and antibiotic discs were placed onto the surface using a sterile applicator to ensure proper contact. The plates were incubated at 37°C for 24 hours. To detect extended-spectrum β-lactamase (ESBL) production, the double-disc synergy test [29]. A total of ten antimicrobial agents were evaluated: ampicillin (AMP), amoxicillin-clavulanate (AMC), cefotaxime (CTX), ceftazidime (CAZ), ceftriaxone (CRO), cefepime (FEP), piperacillin-tazobactam (TZP), ciprofloxacin (CIP), levofloxacin (LEV), and gentamicin (GEN).

Quality control for antimicrobial susceptibility testing was performed using standard reference strains recommended by CLSI guidelines. Reference strains, including *Escherichia coli* ATCC 25922 and *Pseudomonas aeruginosa* ATCC 27853, were used to verify the accuracy and reliability of antibiotic susceptibility testing. Zone diameters obtained for control strains were compared with CLSI standard ranges.

### 2.6. Data analysis

Descriptive statistics, including percentages and 95% confidence intervals (calculated using the binomial proportion method), were used to summarize the prevalence and antimicrobial resistance patterns of Gram-negative bacterial isolates. Graphical representations of the data were prepared using Microsoft Office Excel 2007 to facilitate visual comparison of antimicrobial efficacy across meat types and bacterial species. As this study was based on descriptive statistics, formal statistical comparisons between bacterial species or meat types were not performed, and observed differences should be interpreted cautiously.

## 3. Results

A total of 123 Gram-negative bacterial isolates were recovered from meat samples collected from retail outlets across Sindh, Pakistan. All isolates were rod-shaped by microscopic examination. Cultural characteristics on selective media allowed differentiation of species: *Escherichia coli* and *Klebsiella* spp. produced pink, lactose-fermenting colonies, with *Klebsiella* spp. showing mucoid growth; *Salmonella* spp. and *Citrobacter* spp. formed pale, non-lactose fermenting colonies; *Enterobacter* spp. produced large, sticky, pale pink colonies; *Pseudomonas* spp. displayed smooth, translucent colonies with irregular edges, and *Aeromonas* spp., and formed small, pale colonies.

Among the isolates, *E. coli* was the most prevalent (37.4%, 95% CI: 28.8–45.9), followed by *Klebsiella* spp. (25.2%, 95% CI: 17.5–32.9) and *Salmonella* spp. (17.9%, 95% CI: 11.1–24.7). The remaining isolates comprised Enterobacter spp. (13.8%, 95% CI: 7.7–19.9), Pseudomonas spp. (2.4%, 95% CI: 0–5.2), Citrobacter spp. (2.4%, 95% CI: 0–5.2), and Aeromonas spp. (0.8%, 95% CI: 0–2.4). collectively accounted for 21% of the isolates as shown in Table 1. These findings highlight the diversity of Gram-negative bacteria in retail meat and potential public health risks associated with their consumption.

**Table 1:** Gram-negative bacterial isolates from retail meat samples: Prevalence and colony characteristics.

Bacterial Species	Microscopic Appearance	Colony Characteristics on MacConkey / Nutrient Agar	Number of Isolates	Percentage of Total Isolates (%)	95% CI
*Escherichia coli*	Rods with rounded ends	Pink, lactose-e fermenting colonies	46	37.4	28.8–45.9
*Klebsiella* spp.	Rods with rounded ends	Pink, lactose-fermenting colonies with mucoid growth	31	25.2	17.5–32.9
*Salmonella* spp.	Rods	Non-lactose fermenting, pale colonies	22	17.9	11.1–24.7
*Enterobacter* spp.	Rods	Large, sticky, pale pink colonies	17	13.8	7.7–19.9
*Pseudomonas* spp.	Rods	Smooth, translucent colonies with irregular edges	3	2.4	0-5.2
*Citrobacter* spp.	Rods	Non-lactose fermenting, pale colonies	3	2.4	0-5.2
*Aeromonas* spp.	Straight bacilli	Small, pale colonies on nutrient agar	1	0.8	0–2.4
Total	–	–	123	100	–

Microscopic appearance and colony morphology of Gram-negative isolates were used to differentiate bacterial species. Percentages are calculated based on the total number of isolates (n = 123), with 95% confidence intervals provided.

The IMViC biochemical test series was used to differentiate selected enteric bacteria according to their metabolic characteristics. The observed biochemical profiles were consistent with expected species characteristics. *E. coli* showed the pattern I + , MR + , VP–, C–, whereas *Klebsiella* spp. and *Enterobacter* spp. were typically I–, MR–, VP + , C + . *Salmonella* spp. showed an I–, MR + , VP–, C+ profile. Variable reactions were observed for *Citrobacter* spp. and *Aeromonas* spp. Their responses to the indole, methyl red, Voges-Proskauer, and citrate tests are summarized in Table 2.

**Table 2:** IMViC biochemical test patterns for Gram-negative bacterial isolates.

Organism	Indole (I)	Methyl Red (M)	Voges-Proskauer (V)	Citrate (C)
*Escherichia coli*	+	+	–	–
*Klebsiella* spp.	–	–	+	+
*Salmonella* spp.	–	+	–	+
*Enterobacter* spp.	–	–	+	+
*Pseudomonas* spp.	–	–	–	+
*Citrobacter* spp.	±	+	±	+
*Aeromonas* spp.	+	+	–	±

+ = Positive; – = Negative; ± = Variable depending on strain.

**Fig 1:**
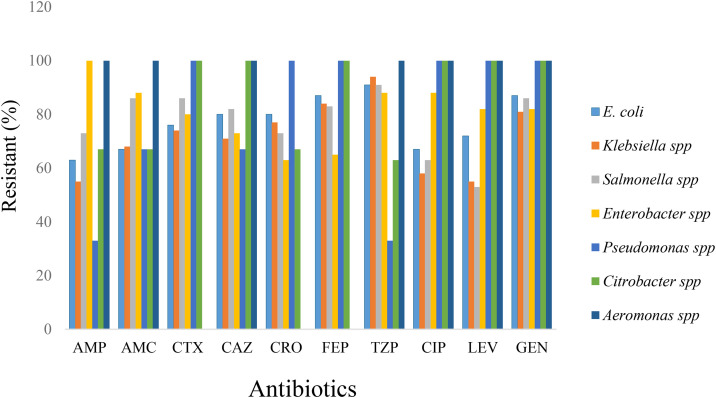
Antibiotic profiles of Gram-negative bacterial isolates from different retail meats.

As presented in Table 3 and Fig 1, bacterial isolates exhibited varying antimicrobial susceptibility patterns, with resistance predominating over susceptibility for most antibiotics tested. *E. coli* showed high resistance to piperacillin–tazobactam (91%), cefepime (87%), gentamicin (87%), ceftazidime (80%), and ceftriaxone (80%). *Klebsiella* spp. similarly demonstrated high resistance, particularly to piperacillin–tazobactam (94%), cefepime (84%), gentamicin (81%), and ceftriaxone (77%). In *Salmonella* spp., the highest resistance rates were observed for amoxicillin–clavulanic acid, cefotaxime, and gentamicin (86% each), followed by ceftazidime and cefepime (82% each).

**Table 3:** Antimicrobial susceptibility patterns of bacterial isolates. Antimicrobial susceptibility patterns of bacterial isolates. Data are presented as the number and percentage of isolates classified as susceptible (S) or resistant (R). No intermediate strains were detected.

Species	Isolates	AMP S	AMP R	AMC S	AMC R	CTX S	CTX R	CAZ S	CAZ R	CRO S	CRO R	FEP S	FEP R	TZP S	TZP R	CIP S	CIP R	LEV S	LEV R	GEN S	GEN R
*E. coli*	46	17/46 (37%)	29/46 (63%)	15/46 (33%)	31/46 (67%)	11/46 (24%)	35/46 (76%)	9/46 (20%)	37/46 (80%)	9/46 (20%)	37/46 (80%)	6/46 (13%)	40/46 (87%)	4/46 (9%)	42/46 (91%)	15/46 (33%)	31/46 (67%)	13/46 (28%)	33/46 (72%)	6/46 (13%)	40/46 (87%)
*Klebsiella* spp.	31	14/31 (45%)	17/31 (55%)	10/31 (32%)	21/31 (68%)	8/31 (26%)	23/31 (74%)	9/31 (29%)	22/31 (71%)	7/31 (23%)	24/31 (77%)	5/31 (16%)	26/31 (84%)	2/31 (6%)	29/31 (94%)	13/31 (42%)	18/31 (58%)	14/31 (45%)	17/31 (55%)	6/31 (19%)	25/31 (81%)
*Salmonella* spp.	22	6/22 (27%)	16/22 (73%)	3/22 (14%)	19/22 (86%)	3/22 (14%)	19/22 (86%)	4/22 (18%)	18/22 (82%)	6/22 (27%)	16/22 (73%)	4/22 (18%)	18/22 (82%)	2/22 (9%)	20/22 (91%)	8/22 (36%)	14/22 (64%)	9/22 (41%)	13/22 (59%)	3/22 (14%)	19/22 (86%)
*Enterobacter* spp.	17	0/17 (0%)	17/17 (100%)	2/17 (12%)	15/17 (88%)	2/17 (12%)	15/17 (88%)	4/17 (24%)	13/17 (76%)	6/17 (35%)	11/17 (65%)	2/17 (12%)	15/17 (88%)	2/17 (12%)	15/17 (88%)	2/17 (12%)	15/17 (88%)	3/17 (18%)	14/17 (82%)	3/17 (18%)	14/17 (82%)
*Pseudomonas* spp.	3	2/3 (67%)	1/3 (33%)	1/3 (33%)	2/3 (67%)	0/3 (0%)	3/3 (100%)	1/3 (33%)	2/3 (67%)	0/3 (0%)	3/3 (100%)	0/3 (0%)	3/3 (100%)	2/3 (67%)	1/3 (33%)	0/3 (0%)	3/3 (100%)	0/3 (0%)	3/3 (100%)	0/3 (0%)	3/3 (100%)
*Citrobacter* spp.	3	1/3 (33%)	2/3 (67%)	1/3 (33%)	2/3 (67%)	0/3 (0%)	3/3 (100%)	0/3 (0%)	3/3 (100%)	1/3 (33%)	2/3 (67%)	0/3 (0%)	3/3 (100%)	1/3 (33%)	2/3 (67%)	0/3 (0%)	3/3 (100%)	0/3 (0%)	3/3 (100%)	0/3 (0%)	3/3 (100%)
*Aeromonas* spp.	1	0/1 (0%)	1/1 (100%)	0/1 (0%)	1/1 (100%)	1/1 (100%)	0/1 (0%)	0/1 (0%)	1/1 (100%)	0/1 (0%)	1/1 (100%)	0/1 (0%)	1/1 (100%)	0/1 (0%)	1/1 (100%)	1/1 (100%)	0/1 (0%)	0/1 (0%)	1/1 (100%)	**0/1 (0%)**	**1/1 (100%)**

**Abbreviations:** AMP, Ampicillin; AMC, Amoxicillin–clavulanic acid (Augmentin); CTX, Cefotaxime; CAZ, Ceftazidime; CRO, Ceftriaxone; FEP, Cefepime; TZP, Piperacillin–tazobactam; CIP, Ciprofloxacin; LEV, Levofloxacin; GEN, Gentamicin; S, susceptible; R, resistant.

*Enterobacter* spp. exhibited complete resistance to ampicillin (100%), with high resistance also recorded for amoxicillin–clavulanic acid, cefotaxime, cefepime, piperacillin–tazobactam, and ciprofloxacin (88% each). Although the number of isolates was small, *Pseudomonas* spp. and *Citrobacter* spp. showed marked resistance to several antibiotics. The single *Aeromonas* isolate was resistant to most tested agents but remained susceptible to cefotaxime and ciprofloxacin. No intermediate-resistant isolates were detected.

Table 4 summarizes the multidrug-resistant (MDR) phenotypes of Gram-negative bacterial isolates and the corresponding antimicrobial classes involved. All *E. coli* isolates exhibited resistance to multiple β-lactam antibiotics, including ampicillin, amoxicillin–clavulanic acid, cefotaxime, ceftazidime, and ceftriaxone, indicating resistance to penicillins/β-lactam combinations and cephalosporins. *Klebsiella* spp. isolates showed resistance to six antibiotics across four classes: β-lactams, cephalosporins, penicillins/β-lactam combinations, and aminoglycosides. *Salmonella* spp. displayed resistance to penicillins, cephalosporins, and fluoroquinolones, while *Enterobacter* spp. exhibited multidrug resistance primarily to penicillins/β-lactam combinations and cephalosporins.

**Table 4:** Multidrug-resistant (MDR) profiles of Gram-negative isolates.

Species	Representative resistance phenotype	Antimicrobial classes involved
*E. coli*	AMP, AMC, CTX, CAZ, CRO	Penicillins/β-lactam combinations; cephalosporins
*Klebsiella* spp.	AMP, AMC, CTX, CAZ, FEP, GEN	Penicillins/β-lactam combinations; cephalosporins; aminoglycosides
*Salmonella* spp.	AMP, AMC, CTX, CIP, LEV	Penicillins/β-lactam combinations; cephalosporins; fluoroquinolones
*Enterobacter* spp.	AMP, AMC, CAZ, CRO, FEP	Penicillins/β-lactam combinations; cephalosporins
*Pseudomonas* spp.	AMP, AMC, GEN	Penicillins/β-lactam combinations; aminoglycosides
*Citrobacter* spp.	CIP, LEV, GEN	Fluoroquinolones; aminoglycosides
*Aeromonas* spp.	CTX, CIP	Cephalosporins; fluoroquinolones

AMP, Ampicillin; AMC, Amoxicillin–Clavulanic acid; CTX, Cefotaxime; CAZ, Ceftazidime; CRO, Ceftriaxone; FEP, Cefepime; TZP, Piperacillin–Tazobactam; CIP, Ciprofloxacin; LEV, Levofloxacin; GEN, Gentamicin; S, Susceptible; and R, Resistant.

Smaller groups of non-fermenting bacteria, including *Pseudomonas* spp., *Citrobacter* spp., and *Aeromonas* spp., also demonstrated multidrug resistance. *Pseudomonas* spp. were resistant to penicillins/β-lactam combinations and aminoglycosides, *Citrobacter* spp. to fluoroquinolones and aminoglycosides, and *Aeromonas* spp. to cephalosporins and fluoroquinolones. These results indicate that multidrug resistance is prevalent across diverse Gram-negative bacteria in retail meat, highlighting the potential for antimicrobial resistance transmission through the food chain.

## 4. Discussion

The rapid rise of antibiotic-resistant bacteria threatens to turn infections that were once readily treatable into life-threatening conditions. Antimicrobial resistance (AMR) was associated with approximately 4.95 million deaths globally in 2019 and continues to impose a substantial public health and economic burden, particularly in developing countries such as Pakistan. In addition to increasing healthcare expenditures and productivity losses, AMR may reduce efficiency in food animal production because of treatment failures and trade-related consequences [30].

In the present study, *Escherichia coli*, *Klebsiella* spp., and *Salmonella* spp. were the most frequently isolated Gram-negative bacteria from retail meat samples collected in Sindh, Pakistan. These findings are consistent with previous reports from Pakistan and other regions showing that retail meat, particularly beef and poultry products, may serve as an important reservoir of Gram-negative foodborne bacteria [3,31]. The detection of these organisms in meat products intended for human consumption raises important concerns for food safety and supports the need for reliable, sensitive, and cost-effective detection methods [32,33].

The biochemical identification profiles observed in this study were consistent with previously reported characteristics for *E. coli*, *Klebsiella* spp., *Salmonella* spp., *Enterobacter* spp., *Pseudomonas* spp., *Citrobacter* spp., and *Aeromonas* spp [34]. The use of IMViC and related biochemical tests, together with culture characteristics and confirmatory methods, allowed practical phenotypic differentiation of the isolates recovered from retail meat samples.

Our findings further demonstrated a high prevalence of antimicrobial resistance among the recovered isolates, particularly among *E. coli*, *Klebsiella* spp., *Salmonella* spp., and *Enterobacter* spp. Resistance was especially common against ampicillin, amoxicillin-clavulanate, and third-generation cephalosporins, indicating substantial selective pressure on commonly used antimicrobial classes [35]. The frequent occurrence of multidrug resistance among these organisms is concerning because Gram-negative pathogens with resistance to multiple antimicrobial classes are increasingly associated with treatment failure, prolonged illness, and reduced therapeutic options in both human and veterinary medicine [36].

The observed resistance patterns are broadly consistent with reports from other low- and middle-income settings, where antimicrobial use in food animal production and weaknesses in regulatory oversight may contribute to the emergence and spread of resistant bacteria [37,38]. Fluoroquinolones and gentamicin retained comparatively better activity against some isolates, although resistance to these agents was still present. This pattern suggests that while some treatment options may remain useful, their effectiveness may be eroding. The detection of multidrug-resistant isolates across multiple bacterial genera, including non-fermenters such as *Pseudomonas* spp. and *Aeromonas* spp., further highlights the possibility of resistance dissemination through the food chain [39].

The public health significance of these findings is considerable. Retail meat is an essential source of nutrition, but contamination during slaughter, processing, transport, storage, or retail handling can expose consumers to antimicrobial-resistant bacteria [10]. Such exposure may result in foodborne infection or facilitate the transfer of resistance determinants to the human gut microbiota. In this context, the presence of resistant Gram-negative bacteria in retail meat reinforces the need for strengthened antimicrobial stewardship, improved slaughterhouse and retail hygiene, enhanced food safety practices, and public education on proper meat handling and cooking [40,41]. These findings also support the importance of a One Health framework that links human, animal, and environmental health in efforts to limit AMR transmission [42].

Several research gaps remain in understanding the full role of retail meat in AMR transmission. Source attribution studies are needed to clarify the contribution of contaminated meat to human colonization and infection, and integrated surveillance systems are needed to track resistance trends across food, animal, and human sectors. Additional work is also required to investigate resistance gene transfer, the occurrence of critical resistance determinants such as carbapenemase- and *mcr*-associated genes, and the influence of non-antibiotic co-selectors such as metals and biocides [43,44].

This study has several limitations that should be considered when interpreting the findings. The sample size was relatively limited and restricted to selected retail outlets in Sindh, which may reduce generalizability to other regions of Pakistan. The study focused on selected Gram-negative bacteria and did not include other relevant foodborne pathogens. In addition, antimicrobial resistance was assessed phenotypically, and the underlying genetic mechanisms of resistance were not characterized. Finally, the small number of isolates for some species, including *Pseudomonas*, *Citrobacter*, and *Aeromonas*, limits the strength of species-specific conclusions. Despite these limitations, the study provides useful baseline evidence that retail meat in Sindh may serve as a reservoir for multidrug-resistant Gram-negative bacteria and supports the need for broader surveillance and molecular investigations in Pakistan.

## 5. Conclusion

Antimicrobial-resistant Gram-negative bacteria in retail meat pose a serious threat to food safety and public health. The high prevalence of resistant isolates, particularly *E. coli*, *Klebsiella* spp., and *Salmonella* spp., highlights the potential for resistant bacteria and their associated resistance determinants to enter the human population through the food chain. These findings emphasize the need for effective surveillance systems, standardized detection methods, improved hygiene throughout meat production and retail distribution, and stronger measures to reduce inappropriate antimicrobial use in food animal production. Policymakers should strengthen regulatory oversight and support coordinated national and international responses to AMR, while consumer education on safe meat handling and cooking remains essential. Future research should expand geographic coverage, include larger sample sizes, and incorporate molecular and genomic approaches to characterize resistance mechanisms and transmission pathways more clearly. A One Health approach integrating human, animal, and environmental health will be essential to limiting the spread of AMR and preserving the effectiveness of existing and future antimicrobial agents.

## Supporting information

S1 File This is the dataset of the supplementary file. (XLSX)
